# Primary purulent bacterial pericarditis due to *Streptococcus intermedius* in the absence of an esophago-pericardial fistula

**DOI:** 10.1093/omcr/omae161

**Published:** 2025-01-18

**Authors:** Sahil M Patel, Varshini Venkatesan, Elaine Simon, Stephen Hull

**Affiliations:** Internal Medicine, Brookwood Baptist Health, 833 Princeton Ave. SW, POB 3, Suite 200E, Birmingham, AL 35211, United States; Internal Medicine, Brookwood Baptist Health, 833 Princeton Ave. SW, POB 3, Suite 200E, Birmingham, AL 35211, United States; Internal Medicine, Brookwood Baptist Health, 833 Princeton Ave. SW, POB 3, Suite 200E, Birmingham, AL 35211, United States; Internal Medicine, Grandview Medical Center, 3690 Grandview Parkway, Birmingham, AL 35243, United States

**Keywords:** pericarditis, esophageal neoplasms, S. intermedius, cardiac tamponade

## Abstract

Purulent bacterial pericarditis is a rare and progressive infection with a high mortality. It is rarely due to *Streptococcus intermedius*, a commensal bacteria found in the oral cavity, gastrointestinal tract, and the genitourinary tract. Here we present a 71-year-old man with history of esophageal adenocarcinoma, status post distal esophagectomy and proximal gastrectomy 2 years prior, who developed cardiac tamponade secondary to primary *S. intermedius* purulent bacterial pericarditis in the absence of an esophago-pericardial fistula. We discuss the clinical manifestations of bacterial pericarditis and pericardial effusions, useful diagnostic tools, and explore possible mechanisms for *S. intermedius* pericarditis infection. This case highlights the importance of prompt suspicion, recognition, and intervention for bacterial pericarditis and cardiac tamponade in the setting of recent esophageal instrumentation. In addition, despite being rare, *S. intermedius* should be recognized as a possible pathogen in purulent bacterial pericarditis even with the absence of an esophago-pericardial fistula.

## Introduction

Pericardial effusions caused by infection can result in bacterial pericarditis and in rare instances, develop purulent bacterial pericarditis, which has a high mortality [1]. The most common bacteria involved include *Streptococci, Staphylococci, Haemophilus,* and *Mycobacterium tuberculosis* [1]. *Streptococcus anginosus* group (SAG), previously known as *S. milleri* group, includes *S. intermedius*, *S. constellatus*, and *S. anginosus* [2]. These commensal bacterial species are naturally found in the oral cavity, gastrointestinal tract, and the genitourinary tract and when compared to other alpha hemolytic streptococci species, have a propensity for more invasive infections [3]. They have a proclivity to form abscesses in the brain, lung, liver, and abdomen, but rarely have led to purulent bacterial pericarditis [2]. Only 7 cases of *S. intermedius* pericarditis have been reported between since 1984 [1, 4]. Most cases of primary SAG bacterial pericarditis are due to direct spread via an esophago-pericardial fistula, which is a dreaded complication of esophageal carcinoma [5]. Here we present a unique case of primary *S. intermedius* purulent bacterial pericarditis in a patient without any evidence of an esophago-pericardial fistula.

## Case report

A 71-year-old man with history of stage IV prostate cancer with bone metastases and esophageal adenocarcinoma status post distal esophagectomy and proximal gastrectomy presented to the emergency department for evaluation of weakness and odynophagia. He reported that his odynophagia may be secondary to his esophageal stent, thus resulting in an inability to tolerate oral intake. He had required numerous esophageal stents over the past 2 years for a recurrent esophageal stricture which developed after his esophageal adenocarcinoma resection. His most recent esophageal stent had been placed 2 months ago. Due to his persistently poor oral intake, he was being evaluated by outpatient surgery for a feeding tube. He had also received whole chest radiation and immunotherapy for his esophageal adenocarcinoma. He denied any dyspnea or chest pain.

**Figure 1:**
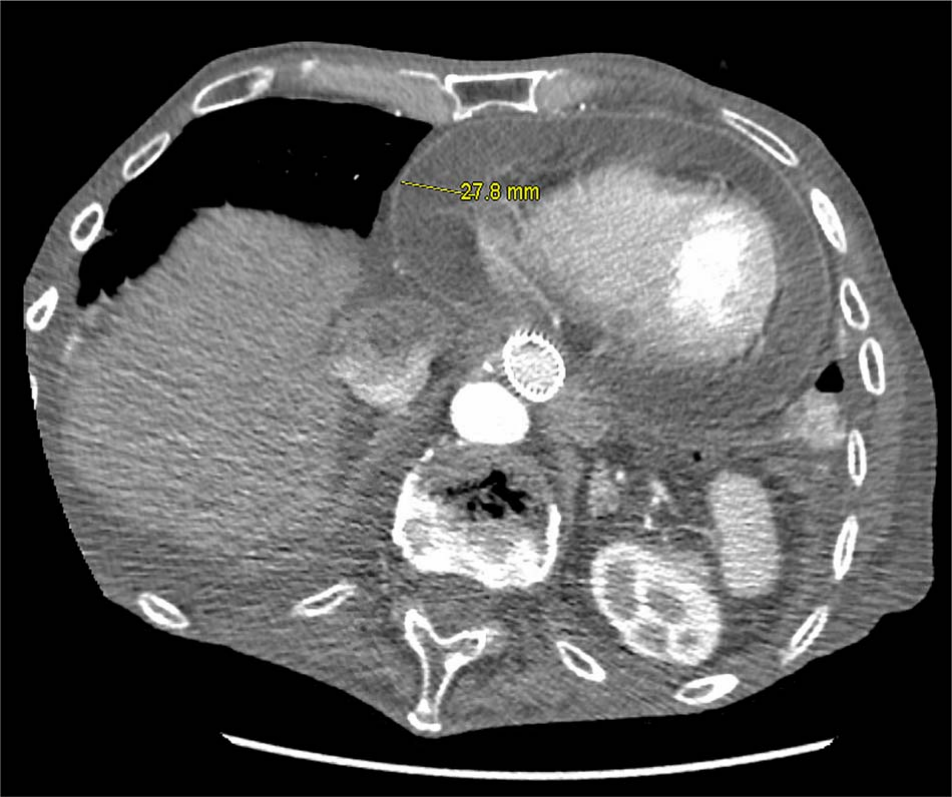
CT scan of the chest showing a large pericardial effusion with maximal thickness of 27.8 mm.

On presentation, his vital signs were significant for temperature 101.8°F, heart rate 104 beats per minute (bpm), and blood pressure 70/54 mmHg. Initial lab work was significant for white blood cell count 13.5 × 10^9^/L, hemoglobin 10.5 g/dL, hematocrit 32.1%, albumin 2.2 g/dL, alkaline phosphatase 306 U/L, and procalcitonin 710 ng/L. Urinalysis showed no signs of infection. Electrocardiogram (EKG) was unremarkable compared to prior EKGs. Computed tomography (CT) of the chest, abdomen, and pelvis showed a large pericardial effusion with concentric smooth rim enhancement concerning for an infectious pericarditis (Fig. 1), bilateral small pleural effusions with bibasilar compression atelectasis and scarring, and a metallic distal esophageal stent with internal air-fluid level. At this time, cardiology and oncology were consulted.

Shortly afterwards, the patient developed a wide-complex tachycardia. An emergent transthoracic echocardiogram redemonstrated a large circumferential pericardial effusion with new evidence of cardiac tamponade. He subsequently underwent emergent bedside pericardiocentesis with removal of 700 cc of purulent fluid with quick improvement in his hemodynamic status. Pericardial fluid was sent to pathology where no malignancy was seen on cytology or cell block, thus raising concern for an infectious etiology. Pericardial fluid gram stain showed many white blood cells, no epithelial cells and no organisms. Pericardial fluid culture was positive for *S. intermedius*. Blood cultures obtained on admission showed no growth at five days. He was started on 2 grams of intravenous ceftriaxone. Once he was stabilized, a CT chest with oral contrast was performed which showed no oral contrast extravasation in the pericardial or pleural space, confirming the absence of an esophageal stent leak or esophago-pericardial fistula. Therefore, we diagnosed him with primary *S. intermedius* purulent bacterial pericarditis in the absence of an esophago-pericardial fistula.

Later on, during his hospitalization, the patient went into cardiac arrest due to ventricular tachycardia. Return of spontaneous circulation was achieved, but given his clinical decline, his family ultimately decided to pursue hospice care. The patient died shortly afterwards.

## Discussion

With the use of broad-spectrum antibiotics, bacterial pericarditis is rare and typically occurs only in immunocompromised patient or those with underlying pericardial disease [6]. It is usually a secondary infection caused by hematogenous dissemination or contiguous spread from surrounding intrathoracic infection, usually from the lung or pleura [1, 7]. Purulent bacterial pericarditis, characterized by frank pus in the pericardial cavity, is a serious manifestation of bacterial pericarditis [1]. Mortality rates approach 100% if it is not identified and treated quickly, but even when identified early, the mortality rate is as high as 40%, likely due to complications like cardiac tamponade, sepsis, and constriction [1, 8, 9].

The classic manifestations of bacterial pericarditis are not always seen as patients present with nonspecific signs and symptoms [1]. Almost all patients present with fever, 25%–37% present with either pleuritic or nonpleuritic chest pain, and less than 50% present with pericardial friction rub and pulsus paradoxus [1, 7]. Lab work can show signs of inflammation, leukocytosis and an elevated troponin in 50% of cases [9, 10]. Cardiomegaly can be seen on chest radiograph along with pleural effusion, pulmonary infiltrates, and mediastinal widening [9]. Approximately 10%–35% of patients have a normal EKG, not showing the classic diffuse ST-segment elevation [7, 9]. Transthoracic echocardiogram is the most sensitive test for pericardial effusions as it can identify evidence of cardiac tamponade, which requires emergent pericardiocentesis [1]. The presence of purulent fluid, however, requires a pericardiocentesis as well. Pericardial fluid should be sent for cell count, Gram, fungal, and acid-fast stain, and culture [1]. Broad-spectrum antibiotics with an antistaphylococcal agent and an aminoglycoside are warranted [1]. This can be narrowed further based on culture and sensitivity results.

With our patient’s negative blood cultures and imaging, he was diagnosed with primary *S. intermedius* purulent bacterial pericarditis. *S. intermedius* is known to spread from the gastrointestinal tract to the pericardial space via esophago-pericardial fistulas. As no evidence for an esophago-pericardial fistula was found, the mechanism behind how *S. intermedius* infected the pericardium of our patient is unclear. Genetic studies have shown that *S. intermedius* has the presence of spreading factors, invasion proteins, adhesions, the Streptococcus invasion locus (Sil) two-component regulator of virulence, and cell wall proteins [3]. However, the possibility that this species evolved to develop a new mechanism of transfer allowing it to be more virulent cannot be ruled out. Given the patient’s immunocompromised status, another hypothesis involves the presence of micro-perforations during his esophageal stenting procedures which possibly led to seeding into the bloodstream. A peculiar finding however, was the lack of any grown on his blood cultures.

This case highlights the importance of prompt suspicion, recognition, and intervention for bacterial pericarditis and cardiac tamponade in the setting of recent esophageal instrumentation. In addition, despite being rare, *S. intermedius* should be recognized as a possible pathogen in purulent bacterial pericarditis even with the absence of an esophago-pericardial fistula.

## Consent

Informed consent for publication was obtained for this case report.

## Guarantor

Stephen D. Hull, Internal Medicine, Grandview Medical Center, 3690 Grandview Parkway, Birmingham, AL. 35 243. United States. Tel: (228) 596-5068. Email: hull.sd@gmail.com

## References

[1] Khan MS, Khan Z, Banglore BS. et al. Primary purulent bacterial pericarditis due to Streptococcus intermedius in an immunocompetent adult: a case report. J Med Case Rep 2018;12:27. 10.1186/s13256-018-1570-x. 29397796 PMC5798186

[2] Faden HS . Infections associated with Streptococcus intermedius in children. Pediatr Infect Dis J 2016;35:1047–8. 10.1097/INF.0000000000001227. 27294306

[3] Denby KJ, Byrne RD, Gomez-Duarte OG. Streptococcus intermedius: an unusual case of purulent pericarditis. Case Rep Infect Dis 2017;2017:5864694. 10.1155/2017/5864694. 28932608 PMC5592407

[4] Christian-Miller N, Goraya S, O'Hayer P. et al. Purulent Streptococcus intermedius pericarditis in the setting of Histoplasma mediastinal lymphadenitis: a case report and literature review. Cureus 2024;16:e62626. 10.7759/cureus.62626. 39027746 PMC11257658

[5] Muto M, Ohtsu A, Boku N. et al. Streptococcus milleri infection and pericardial abscess associated with esophageal carcinoma: report of two cases. Hepato-Gastroenterology 1999;46:1782–4. 10430344

[6] Hall IP . Purulent pericarditis. Postgrad Med J 1989;65:444–8. 10.1136/pgmj.65.765.444. 2690043 PMC2429439

[7] Rubin RH, Moellering RC Jr. Clinical, microbiologic and therapeutic aspects of purulent pericarditis. Am J Med 1975;59:68–78. 10.1016/0002-9343(75)90323-x. 1138554

[8] Keersmaekers T, Elshot SR, Sergeant PT. Primary bacterial pericarditis. Acta Cardiol 2002;57:387–9. 10.2143/AC.57.5.2005459. 12405580

[9] Pankuweit S, Ristic AD, Seferovic PM. et al. Bacterial pericarditis: diagnosis and management. Am J Cardiovasc Drugs 2005;5:103–12. 10.2165/00129784-200505020-00004. 15725041

[10] Bonnefoy E, Godon P, Kirkorian G. et al. Serum cardiac troponin I and ST-segment elevation in patients with acute pericarditis. Eur Heart J 2000;21:832–6. 10.1053/euhj.1999.1907. 10781355

